# A Novel Magnetic Graphene Oxide Composite Absorbent for Removing Trace Residues of Polybrominated Diphenyl Ethers in Water

**DOI:** 10.3390/ma7086028

**Published:** 2014-08-21

**Authors:** Ning Gan, Jiabing Zhang, Shaichai Lin, Nengbing Long, Tianhua Li, Yuting Cao

**Affiliations:** The State Key Laboratory Base of Novel Functional Materials and Preparation Science, Faculty of Material Science and Chemical Engineering of Ningbo University, Ningbo 315211, Zhejiang, China; E-Mails: hcchcnc@126.com (J.Z.); xg1012@126.com (S.L.); longnengbing@nbu.edu.cn (N.L.); litianhua@nbu.edu.cn (T.L.)

**Keywords:** graphene oxide, magnetic disperse solid phase extraction, water treatment, sol-gel technology, polybrominated diphenyl ethers

## Abstract

The purpose of the study was to develop a facile method for the fabrication of a stable and reusable magnetic graphene composite absorbent to remove trace levels of polybrominated diphenyl ethers in water treatment. The poly cationic Fe_3_O_4_@PDDA (poly(diallyldimethyl ammonium chloride) (PDDA)) core-shell structured nanoparticles were first synthesized, and then, DNA was laid on the surface of graphene oxide (GO*x*) to prepare the polyanionic GO*x*@DNA composite. The above materials were then mixed together and adhered together through sol-gel technology. Thus, the Fe_3_O_4_@PDDA/GO*x*@DNA composite absorbent was prepared. Its performance was tested by disperse solid phase extraction and gas chromatography/mass spectrometric (GC/MS) for removing six kinds of indicative polybrominated diphenyl ethers (BDEs) in water samples. The removal percentages of several real samples for six kinds of BDEs (BDE17, BDE28, BDE 71, BDE 47, BDE 66, BDE 100) at the ng/mL order of magnitude were in the range of 88.2%–99.1%. The removal percentage still reached 80.0% when the adsorbent was reused at least 20 times. The results suggested that the magnetic absorbent can obviously remove trace levels of BDEs from large volumes of aqueous solutions in environmental pollution cleanup with high removal efficiency.

## 1. Introduction

Polybrominated biphenyls (BDEs) have been widely applied in many areas, including heat transfer fluids and dielectric fluids [1]. However, they are a typical class of the persistent organic pollutants (POPs) that cause great harm to the environment, as well as human health, due to their toxicity, high stability and biomagnification through the food chain [1]. Because of their low aqueous solubility and bioavailability, BDEs are resistant to biodegradation and are difficult to be removed by conventional physicochemical techniques, such as coagulation, flocculation, sedimentation and filtration [2]. Therefore, it is necessary to remove organic dyes in environmental water. Adsorption has been regarded as one of the most effective physical processes for removing BDEs in wastewaters, since it can produce high quality water and also be a process that is economically feasible. Activated carbon (AC) is the most widely used commercially available absorbent [3]. However, difficult regeneration and lower absorption capacity limits its application in waste water treatment [4].

Carbon nanotubes and graphene oxide (GO*x*) are a class of electron-rich carbon adsorbents with a large delocalized π-electron system, which shows promising applications in water treatment [5]. Due to grapheme oxide’s unique plate structure and properties, including large specific surface area, excellent stability and rich functionalities [6], organic compounds with a benzene ring can be strongly adsorbed on GO*x*. However, a major defect comes from the difficulties that are associated with the separation of water-insoluble GO*x* from water solutions after absorption procedures. Magnetic composite materials also have many advantages, such as simple separation, high adsorption capacity and cyclic utilization, which can greatly expand their application in environmental purification. Magnetic separation has been developed to facilitate the collection of GO*x* [7,8].

Various magnetic materials can be used as adsorbents. Fe_3_O_4_@PDDA (poly(diallyldimethyl ammonium chloride) (PDDA)) with a core-shell structure can form magnetic nanoparticles that reveal excellent dispersion and chemical stability, which are not easy to reunite and for saturation magnetization. The sol-gel technique will be selected as the preparation method for combining Fe_3_O_4_@PDDA with positive charges and GO*x*@DNA, which has a large amount of negative charges [7,8]. In the preparation process, oppositely-charged species are deposited on a carrier in sequences through electrostatic assembly [8]. In this study, the Fe_3_O_4_@PDDA core-shell-structured nanoparticles were modified with GO*x*@DNA through sol-gel technology in order to improve their stability and adsorption capacity. Herein, the magnetic microspheres with Fe_3_O_4_ as the core and PDDA on its surface as a shell were synthesized through the sol-gel approach, which can obtain polycationic magnetic microspheres (Fe_3_O_4_@PDDA). The above materials were modified with polyanionic graphenes, which were modified by negative DNA. In the composites, the polyelectrolyte layer cannot only enhance the adhesive strength between GO*x*@DNA and Fe_3_O_4_@PDDA, but also increase the amount of the GO*x* on it.

Combined with magnetic nanocomposites (Fe_3_O_4_@PDDA/GO*x*@DNA) as adsorbents, the technique of magnetic solid-phase extraction (mSPE) was chosen for an effective pretreatment assay to enrich and purify analytes in samples with a complex matrix [9,10,11,12,13,14,15]. With the magnetic property, the new absorbent using mSPE could avoid time-consuming procedures and be performed directly to pretreat crude samples without the need for centrifugation or filtration, due to the facile magnetic separation [10]. The aim of this study was to develop a simple, rapid, inexpensive and robust methodology to remove BDEs in real water samples. The proposed methodology included magnetic nanocomposites (Fe_3_O_4_@PDDA/GO*x*@DNA) prepared by sol-gel technology for removing BDEs in environmental water samples. The adsorption experiment showed that the magnetic nanocomposites could be reused at least 20 times without a significant loss of the clean-up efficiency. The adsorbents were successfully employed for removing the trace level of BDEs in real water samples.

## 2. Materials and Methods

### 2.1. Chemicals

2,4-Dibromophenyl 2-bromophenyl ether (BDE17), 2,4,4′-tribromodiphenyl ether (BDE28), 2,3′,4′,6-tetrabromodiphenyl ether (BDE 71), 2,2′,4,4′-tetrabromodiphenyl ether (BDE 47), 2,3′,4,4′-tetrabromodiphenyl ether (BDE 66) and 2,2′,4,4′,6-pentabromodiphenyl ether (BDE 100) were obtained from Accu Standard Inc. (New Haven, CT, USA). Ct-DNA (Lot: D4522-1MG, Sigma, New York, NY, USA) and poly(diallyldimethyl ammonium chloride) (PDDA, 20%, w/w, in water, MW = 100,000–200,000) were purchased from Sigma-Aldrich (New York, NY, USA). The carboxyl of graphenes (purity > 95 wt%, ASH < 1.5 wt%, SSA > 500 m^2^/g) were purchased from Nanoport. Co. Ltd. (Shenzhen, China). Ferric chloride (FeCl_3_·6H_2_O), ammonia (28%), *n*-hexane, ethylene glycol, tetraethyl orthosilicate (TEOS), sodium acetate and dichloromethane were purchased from Beijing Chemicals Corporation (Beijing, China). The syringe filters were purchased from Xingya (Shanghai, China). All other chemicals were used as received without further purification. The water in this work was deionized water.

### 2.2. Preparation of Fe_3_O_4_@PDDA/GOx@DNA Nanoparticles

#### 2.2.1. Synthesis of Fe_3_O_4_ Nanospheres

The magnetic nanoparticles were prepared based on the previously reported hydrothermal procedures [11]. 5.75 g FeCl_3_·6H_2_O and 15.33 g of sodium acetate were dissolved in 320 mL of ethylene glycol under mechanical stirring. The obtained homogeneous yellow solution was transferred to a Teflon-lined stainless steel autoclave, sealed and heated at 200 °C for 8 h. The obtained black magnetite precipitate was washed with ethanol and deionized water three times, then dried at 60 °C under vacuum.

#### 2.2.2. Synthesis of Fe_3_O_4_@PDDA Nanospheres

The Fe_3_O_4_@PDDA nanospheres were prepared according to the previously reported sol-gel method [12]. Briefly, 0.5 g of dry Fe_3_O_4_ particles were firstly pretreated with 0.1 M HCl aqueous solution (100 mL) by ultrasonication for 15 min. Moreover, pH 7.0 PBS was added to maintain the neutral characteristics. The resulting suspension was stirred in 0.1 mol/L PDDA for 6 h at room temperature. The obtained Fe_3_O_4_@PDDA nanospheres were magnetically separated, washed with ethanol and deionized water, then dried at 60 °C in vacuum for 2 h. The black precipitate was washed with deionized water three times and dried at 60 °C in vacuum for 2 h. 

#### 2.2.3. Synthesis of Fe_3_O_4_@PDDA/GO*x*@DNA Nanoparticles

Graphene was firstly purified in 0.5 M HCl solution by ultrasonication for 4 h. The resulting material was washed with water several times and dried under vacuum at 60 °C overnight [13]. 50 mg purified GO*x* were dispersed in sodium chloride aqueous solution (0.5 mol/L, 100 mL). Then, 6 mL of DNA solution (20%) were added dropwise under vigorous stirring. The mixture was centrifuged at 5000 rpm for 5 min and washed with deionized water to remove the superfluous DNA. The obtained GO*x*/DNA with a positive charge was dried at 60 °C in vacuum overnight. 

**Figure 1:**
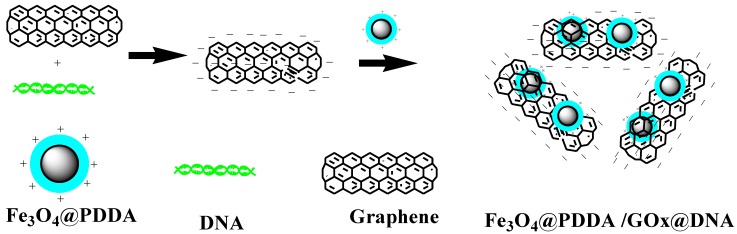
Synthesis strategy of Fe_3_O_4_@PDDA /GO*x*@DNA.

Fifty milligrams of Fe_3_O_4_@PDDA nanoparticles with a negative charge were dispersed in 100 mL of water. Then, 50 mg of dry GO*x*/DNA were added with mechanical stirring. After reaction for 60 min, the product was collected with a magnet, then washed with ethanol and deionized water, dried at 60 °C in vacuum for 2 h. The preparation process is shown in Figure 1.

### 2.3. The Removal Experiment for BDEs in Waters

Sample solutions (1000 ng/mL) with six kinds of BDEs were prepared in isooctane and stored at 4 °C. All of the working solutions of BDEs were 100 ng/mL and prepared daily by appropriate dilution of the stock solutions with *n*-hexane. All glassware used in the study was soaked and washed in acetone, then rinsed with *n*-hexane and dried at 100 °C for 4 h.

**Figure 2:**
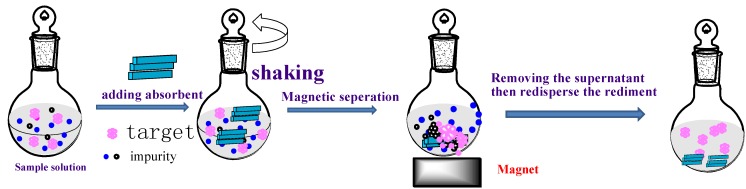
The absorption and detection process to remove the polybrominated biphenyls (BDEs) in water.

For the adsorption steps, 30 mg of the adsorbents (Fe_3_O_4_@PDDA/GO*x*@DNA) were rinsed and activated in 1 mL of methanol, then uniformly dispersed into the 100-mL filtered water sample in a beaker (100 mL of deionized water spiked with 10 ng/mL of BDEs were used, and all of the experiments were performed in triplicate). The mixture was stirred vigorously at 300 rpm for 30 min. Subsequently, a strong magnet was placed at the bottom of the beaker, so that the adsorbents were isolated from the solution. The solution became limpid after about 1 min. The procedures are shown in Figure 2.

For the desorption step, firstly, the supernatant was decanted carefully with the magnet, and the pre-concentrated analytes were transferred to the syringes of an organic membrane (0.45 μm) and dried at 60 °C under vacuum for 4 h. Secondly, the adsorbed compounds were eluted from the adsorbents with 10 mL of a mixture of *n*-hexane:dichloromethane (40/60, v/v) as the elute. The flow rate was controlled at 2 mL/min, and the eluates were collected with a centrifugal tub. Thirdly, the adsorbents were dried with nitrogen to remove the dichloromethane and *n*-hexane. Then, the residue was dissolved in 1 mL of *n*-hexane with 0.5 mL of concentrated sulfuric acid to further acidification. Finally, the mixed solution was centrifuged for 10 min, and the supernatant liquid was stored in a fridge overnight. One microliter of this solution was injected into the GC/MS system for analysis. The experiment process is shown in Figure 2. The overall procedure took place at room temperature. The BDE removal percentage was calculated using Equation (1) [16].

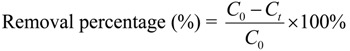
(1)
where *C*_0_ is the original concentration of BDEs and *C_t_ r*epresents the residual concentration of BDEs after extraction, respectively.

### 2.4. Real Sample Preparation

Water samples were collected in Ningbo in September, 2013. Tap water samples were taken from our laboratory in Yongjiang River, and water samples were acquired from Jiangbei District (Ningbo). All samples were collected randomly and filtered through 0.45-μm membranes to remove suspended particles.

## 3. Results and Discussion

### 3.1. Characterization of Adsorbents

**Figure 3:**
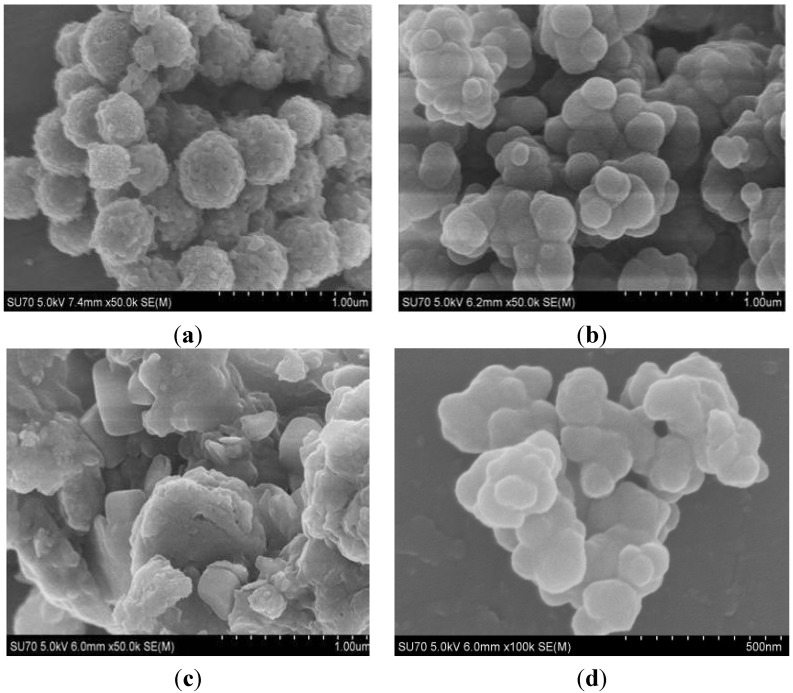
SEM images of: (**a**) Fe_3_O_4_; (**b**) Fe_3_O_4_@PDDA; (**c**) GO*x*@DNA; (**d**) Fe_3_O_4_@PDDA/GO*x*@DNA.

SEM figures were used to characterize the morphology of Fe_3_O_4_, Fe_3_O_4_@PDDA, GO*x*@DNA and Fe_3_O_4_@PDDA/GO*x*@DNA (Figure 3a–d). Figure 3a shows that the mean particle size of Fe_3_O_4_ NPs was about 300 nm. Figure 3b shows the Fe_3_O_4_@PDDA cross-linked together, which proved that PDDA as a polymer was successfully coated onto Fe_3_O_4_ NPs. The tree-like structure of SEM images (Figure 3c) of GO*x*@DNA showed that GO*x*@DNA was successfully synthesized. Both the feathery and spherical composite structures in Figure 3d also proved that GO*x*@DNA was successfully absorbed on the surface of Fe_3_O_4_@PDDA through sol-gel technology.

**Figure 4:**
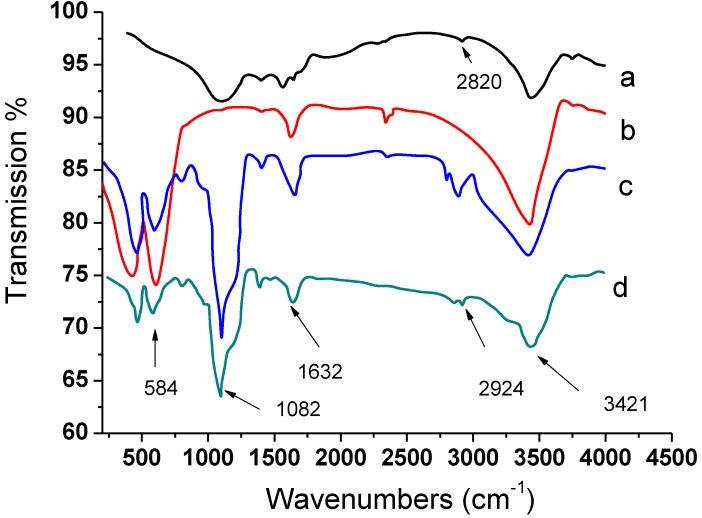
IR spectra of: (**a**) GO*x*; (**b**) Fe_3_O_4_; (**c**) Fe_3_O_4_@PDDA; (**d**) Fe_3_O_4_@PDDA/GO*x*@DNA.

Figure 4 displayed the IR spectra of GO*x*, Fe_3_O_4_, Fe_3_O_4_@PDDA and Fe_3_O_4_@PDDA/GO*x*@DNA. Strong absorption bands between 3300 and 3500 cm^−1^ were due to O–H stretching vibration. The types of peaks shown by GO*x* were between 1300 and 1100 cm^−1^, which were due to the stretching of phenyl-carbonyl C–C bonds. The 2924 and 2820 cm^−1^ bands were due to C–H (Figure 4a). As shown in Figure 4b, the peaks at 584 and 467 cm^−1^ were the stretching vibration due to the Fe–O bonds. Compared to the two spectra (Figure 4b,c), the Fe–O peaks shift to 580 and 473 cm^−1^ (Figure 4c). From Figure 4c,d, we could see that the C–H stretching vibrating band of Fe_3_O_4_@PDDA/GO*x*@DNA at 2924 and 2820 cm^−1^ appeared. All of these demonstrated that GO*x* was successfully modified to the Fe_3_O_4_@PDDA through sol-gel technology.

**Figure 5:**
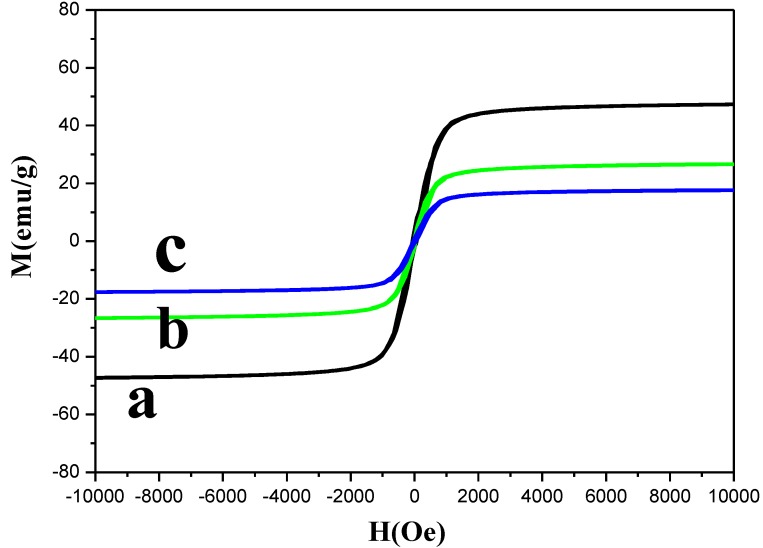
Vibrating sample magnetometer (VSM) magnetization curves of: (**a**) Fe_3_O_4_; (**b**) Fe_3_O_4_@PDDA; (**c**) Fe_3_O_4_@PDDA/GO*x*@DNA.

Figure 5 shows the magnetization curves of Fe_3_O_4_, Fe_3_O_4_@PDDA and Fe_3_O_4_@PDDA/GO*x*@DNA at room temperature. Maximum saturation magnetization of Fe_3_O_4_@PDDA was measured at 25.4 emu/g, which was lower than that of magnetic Fe_3_O_4_ alone (48.2 emu/g). This might be due to the nonmagnetic surface of the PDDA. The saturation magnetization of Fe_3_O_4_@PDDA/GO*x*@DNA was at 18.2 emu/g. Although the addition of the nonmagnetic part (GO*x*) led to a decrease in the saturation magnetizations, the obtained Fe_3_O_4_@PDDA/GO*x*@DNA still had a high saturation magnetization. It was observed that Fe_3_O_4_@PDDA/GO*x*@DNA adsorbents could be dispersed into water solution readily. Thus, the magnetic adsorbents loaded with analytes could be isolated from the matrix conveniently by using an external magnet when necessary.

**Figure 6:**
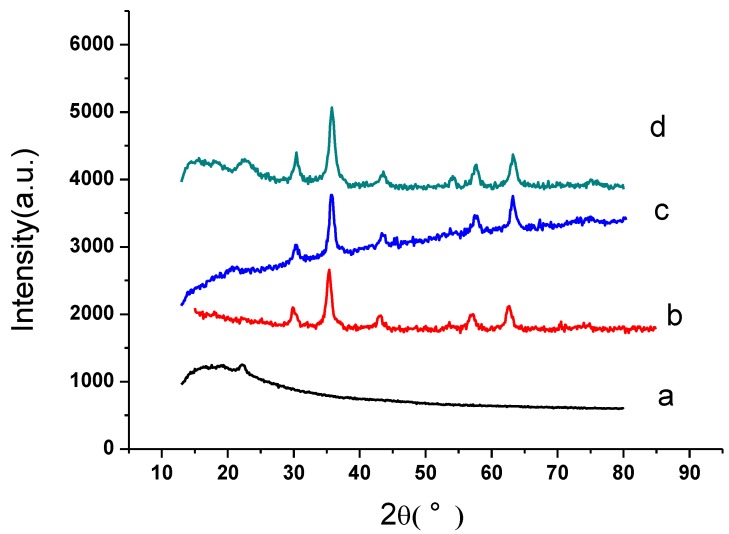
X-ray diffraction (XRD) patterns of: (**a**) GO*x*; (**b**) Fe_3_O_4_; (**c**) Fe_3_O_4_@PDDA; (**d**) Fe_3_O_4_@PDDA/GO*x*@DNA.

The XRD pattern in Figure 6a–d showed the crystal structure of the materials, GO*x*, Fe_3_O_4_, Fe_3_O_4_@PDDA and Fe_3_O_4_@PDDA/GO*x*@DNA, respectively. The well-resolved diffraction peak revealed the crystallinity of the GO*x*, which was located at 2θ = 22.5°. There were several relatively strong diffraction peaks at 2θ = 30.2°, 35.5°, 43.3°, 57.3° and 62.7° in Figure 6b, which were quite similar to those of the Fe_3_O_4_ nanoparticles reported by other groups [14]. From the XRD pattern of Fe_3_O_4_@PDDA/GO*x*@DNA in Figure 6e, the main characteristic peaks of Fe_3_O_4_ still remained and the strong diffraction peak at 2θ = 22.5° (GO*x*) appeared again, which indicated that the composite was composed of Fe_3_O_4_@PDDA and GO*x*@DNA. 

### 3.2. Kinetic Study

**Figure 7:**
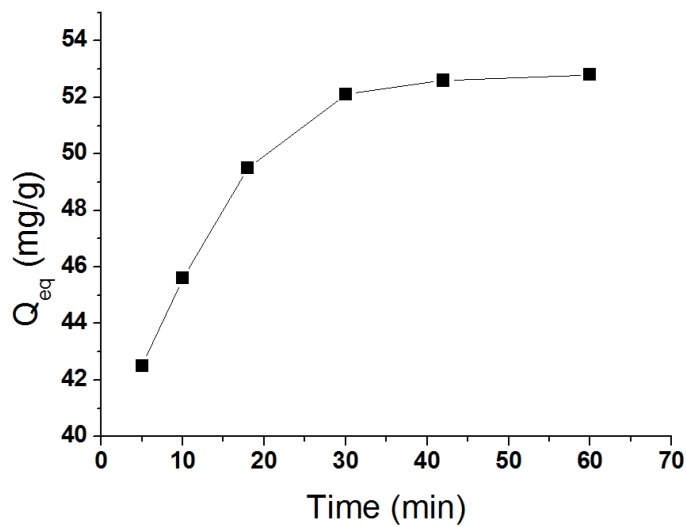
The effect of adsorption time on the adsorption capacity.

As shown in Figure 7a, the kinetics of the uptake of BDE28 by the Fe_3_O_4_@PDDA/GO*x*@DNA (100 mL of deionized water spiked with 152 ng/mL onto 30 mg of the adsorbents) was studied from the adsorption rate of BDE28 to the adsorbents, which showed two stages, a quick stage at the beginning and a slow stage before the equilibrium. The results (Figure 7a) showed that the rate of adsorption for BDE28 was very rapid in the first 20 min; thereafter, it decreased gradually, until it reached a plateau after 30 min, indicating the equilibrium of the system. The phenomena of the adsorption kinetics may be due to the adsorption of BDE28 on the exterior surface of the adsorbent at the initial period of contact time. When the adsorption on the exterior surface reached the saturation point, BDE28 reached into the pores of the adsorbent. In order to elucidate the adsorption process, the results were fitted using the Lagergren first-order model [17] and the pseudo second-order model [18]. The Lagergren first-order model can be expressed as follows:

ln (*Q_eq_* − *Q_t_*) = ln *Q*_1_ − *k*_1 _*t* (2)

where *k* (min^−1^) is the rate constant of adsorption and *Q_t_* (mg g^−1^) and *Q_eq_* (mg g^−1^) represent the amount of the dye adsorbed at any time *t* (min) and at equilibrium, respectively. The rate constant *k* can be determined from the slope of the plot obtained by plotting ln (*Q_eq_* − *Q_t_*) *versus* *t*.

The pseudo-second-order model equation is given as:

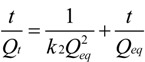
(3)
where *k*_1_ (min^−1^) and *k*_2_ (g mg^−1^ min^−1^) are the rate constant of the Lagergren first-order model and pseudo-second-order model, *Q_t_* (mg g^−1^) and *Q_eq_* (mg g^−1^) represent the amount of the dye adsorbed at any time *t* (min) and at equilibrium and *Q*_1_ (mg g^−1^) and *Q*_2_ (mg g^−1^) are the calculated adsorption capacity of the Lagergren first-order model and pseudo-second-order model.

The Lagergren first-order rate constant *k*_1_ and *Q*_1_ can be determined from the intercept and slope of the plot obtained by plotting ln (*Q_eq_* − *Q_t_*) *versus* t; the pseudo-second-order rate constant *k*_2_ and *Q*_2_ can be determined from the intercept and slope of the plot obtained by plotting *t*/*Q_t_ versus* *t*.

The calculated parameters for Lagergren first-order model and pseudo-second-order model and the correlation coefficients (*r*^2^) are listed in Table 1.

**Table 1:** Kinetic parameters for adsorption of BDE28 onto Fe_3_O_4_@PDDA/GO*x*@DNA.

Sample	*Q*_eq_ (mg g^−1^)	Lagergren First-Order Model			Pseud Second-Order Model		
		*Q*_1_ (mg g^−1^)	*K*_1_ (min^−1^)	*r* ^2^	*Q*_2_ (mg g^−1^)	*k*_2_ (g mg^−1^ min^−1^)	*r* ^2^
BDE28	49.1	49.0	0.387	0.998	49.1	0.023	0.984

A comparison of *Q_eq_* data (Table 2) shows that the calculated *Q*_2_ values of the pseudo-second-order equation are generally closer to the experimental *Q_eq_* values compared to the calculated *Q*_1_ values of the Lagergren first-order equation. The correlation coefficients for the first order kinetic model obtained for all of the studied BDE28 were above 0.998. Therefore, the adsorption process studied follows the Lagergren first-order model better. Based on the assumption of the pseudo-second-order model [18], it can be concluded from the experimental result that the adsorption of cationic BDE28 on G-SO_3_H/Fe_3_O_4_ is mainly due to chemical adsorption [19].

**Table 2:** Removal percentages of six kinds of BDEs from real water samples spiked with target analytes.

Analytes		BDE17	BDE28	BDE71	BDE47	BDE66	BDE100
Tap water	Found (ng/mL)	ND	ND	ND	ND	ND	ND
	Removal percentages (%) (Add 5 ng/mL)	92.5 ± 2.3	94.1 ± 3.1	87.8 ± 2.9	93.2 ± 2.8	93.3 ± 3.9	90.2 ± 3.4
	Removal percentages (%) (Add 5 ng/mL)	92.3 ± 1.8	97.1 ± 1.3	95.2 ± 3.2	95.3 ± 3.1	88.9 ± 4.2	89.7 ± 3.2
Zhenhai River Water 1	Found (ng/mL)	1.62	1.53	1.42	ND	ND	ND
	Removal percentages (%) (Add 5 ng/mL)	90.2 ± 3.4	93.2 ± 4.8	92.1 ± 4.3	93.4 ± 3.2	89.5 ± 3.5	90.4 ± 3.3
	Removal percentages (%) (Add 5 ng/mL)	89.6 ± 1.8	89.7 ± 3.4	90.6 ± 3.4	90.7 ± 2.1	98.8 ± 3.4	93.5 ± 3.2
Yongjiang River water	Found (ng/mL)	1.42	1.21	ND	ND	ND	ND
	Removal percentages (%) (Add 5 ng/mL)	92.8 ± 2.1	97.5 ± 1.2	93.3 ± 3.2	92.4 ± 4.3	94.1 ± 3.1	93.3 ± 1.7
	Removal percentages (%) (Add 5 ng/mL)	92.3 ± 2.8	99.5 ± 2.9	94.1 ± 5.8	92.5 ± 4.7	92.3 ± 4.9	89.1 ± 2.8

ND: not detected.

### 3.3. Adsorption Isotherms

The adsorption isotherm gave the most significant information, which pointed out how the adsorbate molecules were distributed between the liquid phase and solid phase when the adsorption process reaches equilibrium. The adsorption isotherm helped to disclose the adsorption mechanism of BDE28 onto Fe_3_O_4_@PDDA/GO*x*@DNA. According to previous reports, the stronger adsorption of aromatic compounds on GOx was attributed to the π–π interaction. In addition, the hydrophobic effect was another important factor that was responsible for the adsorption on GO*x*. The adsorption of BDE28 on Fe_3_O_4_@PDDA/GO*x*@DNA was studied under ambient conditions using a batch technique. In the removal of BDE28 from aqueous solutions, the amount of adsorbents used in the cleaning procedure was vital for the economic application. In our study, the experimental data of BDE28 were fitted by employing the Langmuir and Freundlich model [20,21,22].

The form of the Langmuir isotherm can be represented by the following equation:

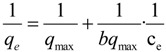
(4)


**Figure 8:**
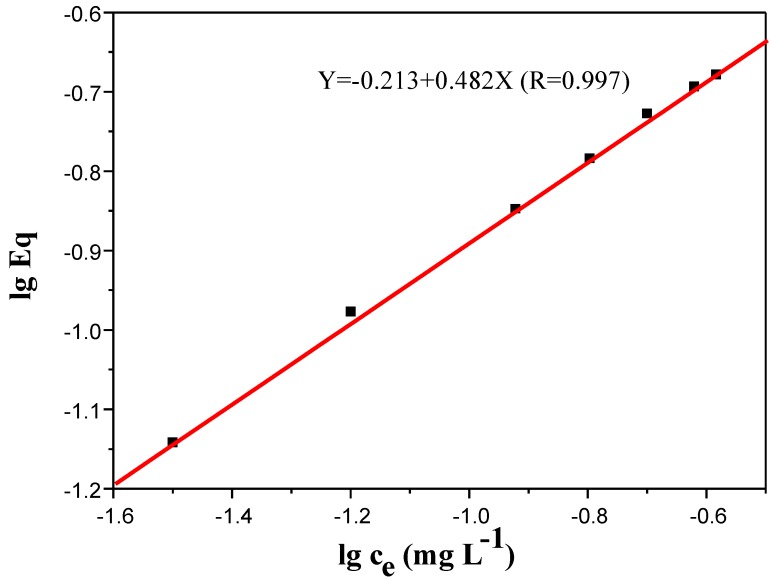
Freundlich isotherm for the adsorption of BDE28 to Fe_3_O_4_@PDDA/GO*x*@DNA.

Here, *q*_max_ and *b* were Langmuir constants related to the adsorption capacity and adsorption energy, respectively. The standard correlation coefficient (*R*^2^) was employed to compare the goodness of fit between model prediction and experimental data. The calculated values of *q*_max_ and 1/*b* were 49.1 mg/g and 4.2, respectively; *R*^2^ was 0.998. The Freundlich isotherm model is expressed as follows:

log *q_e_* = log *k_F_* + *n*log *c_e_* (5)

where *k_F_* represents the adsorption capacity when the adsorbate equilibrium concentration equals one and *n* represents the degree of adsorption dependence at the equilibrium concentration. The experimental data was not in good agreement with the Freundlich isotherm model after calculation (Figure 8). The absorbent had a high nitrogen BET specific area of ~310 m^2^/g (calculated in the linear relative pressure range from 0.1 to 0.3) [22]. The average pore size was 3 nm, which showed it was microporous.

### 3.4. Effect of Adsorption Conditions

In this experiment, several parameters, including adsorption conditions and desorption conditions, were discussed to achieve the best removal efficiency for BDEs. To study the extraction performance of the mSPE under different experimental conditions, 100 mL of deionized water spiked with 10 ng/mL of BDEs were used, and all of the experiments were performed in triplicate. The results were used as a means of optimization. 

#### 3.4.1. Adsorption Time

**Figure 9:**
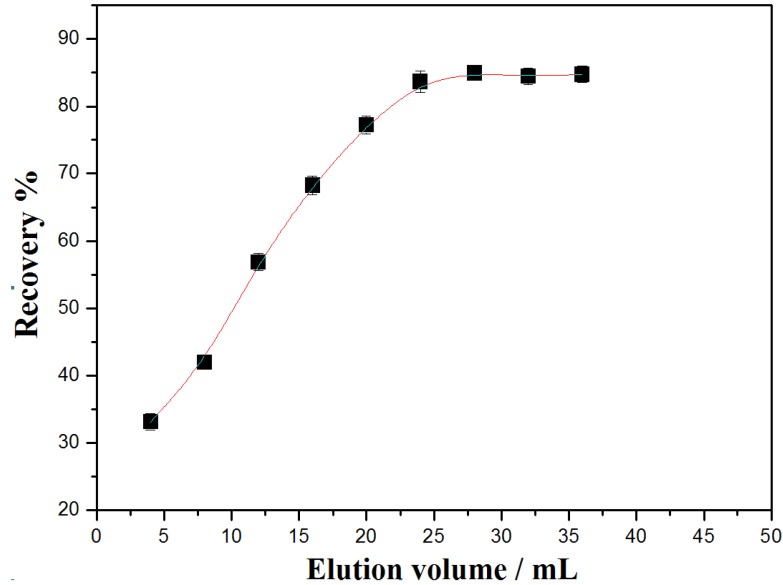
The effect of the adsorption time on the adsorbed amount of BDE28 by Fe_3_O_4_@PDDA/GO*x*@DNA.

In order to assess the ability of the Fe_3_O_4_@PDDA/GO*x*@DNA to adsorb BDE28, equilibrium adsorption time profiles were derived by increasing the adsorption time of 100 mL of the water sample. The results showed that there was a rapid removal efficiency when the adsorption time increased from 5 to 50 min. After 30 min, the maximum removal percentages were obtained for BDE28, which were close to 90% (Figure 9). Consequently, an adsorption time of 30 min was selected for further study.

#### 3.4.2. Amount of the Adsorbents

**Figure 10:**
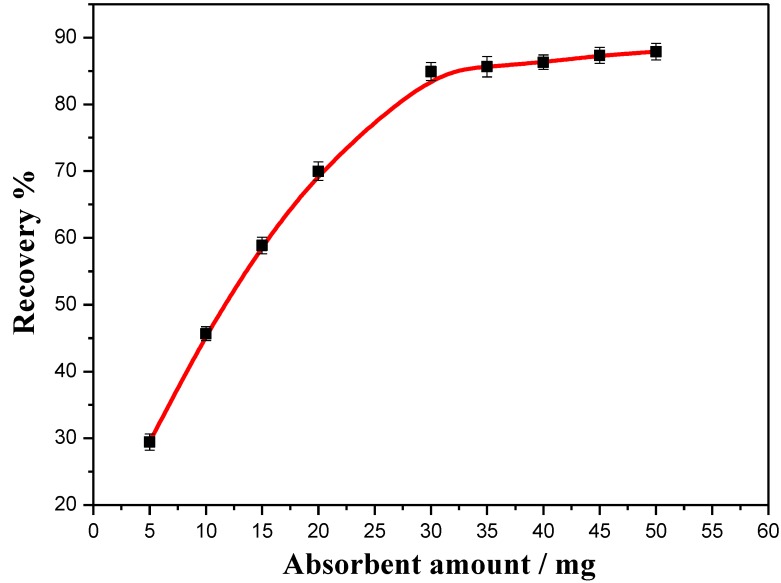
The effect of the mount of the adsorbents on the recovery of BDE28 by Fe_3_O_4_@PDDA/GO*x*@DNA.

To achieve the highest maximum removal percentages towards the target analytes, the amount of Fe_3_O_4_@PDDA/GO*x*@DNA was increased from 5 mg to 50 mg. The overall recoveries were augmented significantly when the amount of adsorbents increased up to 20 mg; when the amount of adsorbents achieved 30 mg (Figure 10), the removal percentages of BDEs were exceeded by 85%.

The overall removal percentages were made to remain unchanged by further increasing the amount of the adsorbents, indicating the remarkable enrichment ability of Fe_3_O_4_@PDDA/GO*x*@DNA as adsorbents. Thirty milligrams of the adsorbents were employed in the study by considering the sensitivity and consumption of the material.

#### 3.4.3. Effect of the Solution pH

**Figure 11:**
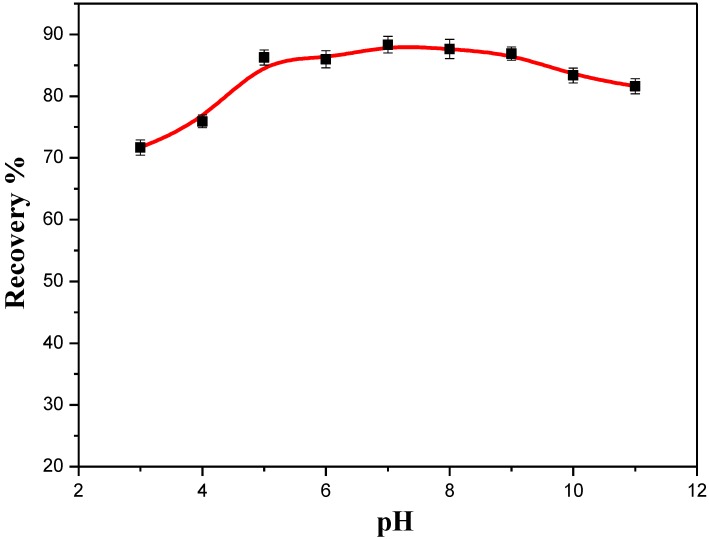
The effect of pH on the recovery of BDE28 by Fe_3_O_4_@PDDA/GO*x*@DNA.

The pH was adjusted between 3.0 and 11.0 by the addition of 0.1 M HCl or NaOH. The adsorption percentage of BDEs on Fe_3_O_4_@PDDA/GO*x*@DNA fluctuated very little with the recovery ranging from 80% to 85% in a pH range of 5–10, with a lower recovery between pH 2 to 3 (Figure 11). All of this suggested that the analytes could efficiently be adsorbed onto the adsorbent at any pH of the real neutral aqueous solutions. Since the pH of the real water samples was generally in the range 5.0–8.0, there was no need to adjust the pH of the sample solution before adsorption. As seen from our experiment, the absorbent was suitable for PCB pollution cleanup in real work.

### 3.5. The Reusability of the Absorbent

**Figure 12:**
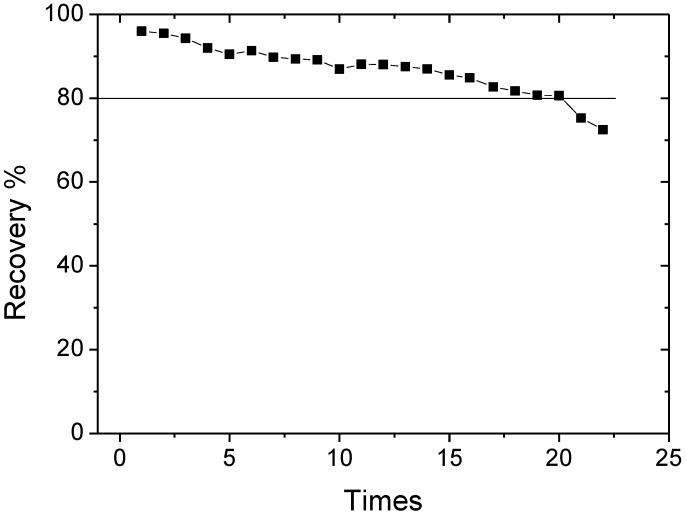
The recycling efficiency *vs.* cycle number after removing BDE28 by Fe_3_O_4_@PDDA/GO*x*@DNA.

The recycling of the nanoparticle adsorbents was studied to verify the chemical stability of the magnetic graphene composites. The Fe_3_O_4_@PDDA/GO*x*@DNA was prepared by a self-assembly technique, and the Fe_3_O_4_@PDDA/GO*x* was prepared by *in situ* synthesis. The adsorbents used in the adsorption procedure were treated with 2 mL of dichloromethane by ultrasonication for 10 min and then washed by 2 mL of *n*-hexane before being applied to the next adsorption procedure. The Fe_3_O_4_@PDDA/GO*x*@DNA adsorbents could be reused 20 times without losing much of the removal percentages of analytes (>80%, Figure 12). However, we found that if the absorbent used Fe_3_O_4_@SiO_2_ as the carrier, it can be reused for at least 50 times, because it has good dispersibility.

### 3.6. The Application of Adsorbent in Environmental Water Samples

**Figure 13:**
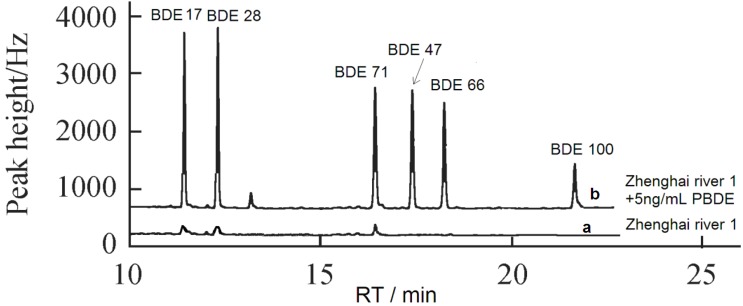
Chromatographs of (**a**) Zhenhai River 1 sample and (**b**) the river sample spiked with 5 ng/mL of BDEs.

To test the removal efficiency of Fe_3_O_4_@PDDA/GO*x*@DNA, tap water, Zhenhai River 1 and its spiked water samples (spiked with 5 ng/mL of BDEs), as well as Yongjiang River water were used as the analysis object by this paper. As shown in Table 2 and Figure 13, the results indicated that Zhenhai River water was contaminated by BDE 17, BDE28 and BDE 71 (Figure 13). The data of spiked water samples showed that Fe_3_O_4_@PDDA/GO*x*@DNA was a suitable material to remove BDEs in environmental water samples. The conventional method of C18 SPE (solid phase extraction with a C18 membrane) was used in our experiment, and the result implied that the adsorbents have excellent removal efficiency (Table 3).

**Table 3:** The detection results of BDEs in Zhenhai River 2 water by the conventional method of C18 SPE and the proposed adsorbent in our manuscript.

Analytes in Zhenhai River 2		BDE17	BDE28	BDE71	BDE47	BDE66	BDE100
C18-SPE	Found (ng/mL)	0.50	0.58	0.69	ND	ND	ND
This work adsorbent	Found (ng/mL)	0.55	0.54	0.78	ND	ND	ND

## 4. Conclusions

In the present work, novel magnetic nanocomposites (Fe_3_O_4_@PDDA@DNA/GO*x*), using the facile layer-by-layer self-assembly method, were used as the adsorbents for removing six kinds of BDEs at the ng/mL order of magnitude in water samples. The method offered high specific removing efficiency to trace levels of BDEs and ease of operation by magnetic separation. More importantly, the magnetic nanocomposites have stable chemical properties and good reusability, which can be reused at least 20 times.
